# Construction and application of a nursing human resource allocation model based on the case mix index

**DOI:** 10.1186/s12912-023-01632-y

**Published:** 2023-12-06

**Authors:** Yanying Yang, Mei He, Yuwei Yang, Qiong Liu, Hongmei Liu, Xi Chen, Wanchen Wu, Jing Yang

**Affiliations:** 1 Department of Hepatobiliary Surgery, Mianyang Central Hospital, School of Medicine, University of Electronic Science and Technology of China, Mianyang, 621000 People’s Republic of China; 2 Department of Laboratory Medicine, Mianyang Central Hospital, School of Medicine, University of Electronic Science and Technology of China, Mianyang, 621000 People’s Republic of China; 3 Nursing Department, Mianyang Central Hospital, School of Medicine, University of Electronic Science and Technology of China, Mianyang, 621000 People’s Republic of China

**Keywords:** Case mix index, Allocation of nursing resources, Daily nursing worktime

## Abstract

**Background:**

The case mix index (CMI) may reflect the severity of disease and the difficulty of care objectively, and is expected to be an ideal indicator for assessing the nursing workload. The purpose of this study was to explore the quantitative relationship between daily nursing worktime (DNW) and CMI to provide a method for the rational allocation of nursing human resources.

**Methods:**

Two hundred and seventy-one inpatients and 36 nurses of the department of hepatobiliary surgery were prospectively included consecutively from August to September 2022. The DNW of each patient were accurately measured, and the CMI data of each patient were extracted. Among 10 curve estimations, the optimal quantitative model was selected for constructing the nursing human resource allocation model. Finally, the applicability of the allocation model was preliminarily assessed by analyzing the relationship between the relative gap in nursing human resources and patient satisfaction, as well as the incidence of adverse events in 17 clinical departments.

**Results:**

The median (P25, P75) CMI of the 271 inpatients was 2.62 (0.92, 4.07), which varied by disease type (*F* = 3028.456, *P* < 0.001), but not by patient gender (*F* = 0.481, *P* = 0.488), age (*F* = 2.922, *P* = 0.089), or level of care (*F* = 0.096, *P* = 0.757). The median (P25, P75) direct and indirect DNW were 76.07 (57.98, 98.85) min and 43.42 (39.42, 46.72) min, respectively. Among the 10 bivariate models, the quadratic model established the optimal quantitative relationship between CMI and DNW; DNW = 92.3 + 4.8*CMI + 2.4*CMI^2^ (*R*^2^ = 0.627, *F* = 225.1, *p* < 0.001). The relative gap between theoretical and actual nurse staffing in the 17 clinical departments were linearly associated with both patient satisfaction (*r* = 0.653, *P* = 0.006) and incidence of adverse events (*r* = − 0.567, *P* = 0.021). However, after adjusting for other factors, it was partially correlated only with patient satisfaction (r_partial_ = 0.636, *P* = 0.026).

**Conclusion:**

The DNW derived from CMI can be used to allocate nursing human resources in a rational and convenient way, improving patient satisfaction while ensuring quality and safety.

**Supplementary Information:**

The online version contains supplementary material available at 10.1186/s12912-023-01632-y.

## Introduction

Allocation of nursing human resource refers to the number and type of nurses required to provide patient care in a health care facility, with the aim of matching a certain nursing workload with the appropriate nursing staff to meet the demands for nursing care of patients [1, 2]. A growing number of studies worldwide have confirmed that allocation of nursing human resources is closely related to patient mortality, infection rates, quality of care, and duration of hospitalization, among others [3, 4]. This highlights the importance of utilizing scientifically sound methods for allocating nursing manpower.

Researchers abroad [5] tended to investigate the on-demand allocation of nursing human resources from three perspectives: patient dependency classification, disease severity grading, and nursing intensity measurement. In the United States, nursing human resources are allocated based on the demand in actual utilization of nursing services by residents. In Canada [6], a Project Research in Nursing (PRN) method is used to calculate the total number of nursing hours for various activities in a nursing unit within 24 hours, then nursing human resources are allocated. In 1978, the Ministry of Health (now the National Health Commission) of China drafted the *Principles for the Organization of General Hospitals (Trial Draft)*, which proposed that the ratio of the number of ward beds to nursing staff should be 1:0.4 when setting nursing positions [7], and this ratio is used to this day. However, with the extension of the scope and connotations of nursing and the complexity of patient care, the current calculation of nursing human resources by ward bed-to-nurse ratio fails to accurately reflect nurses’ workload. Therefore, it is necessary to explore more reasonable methods for allocating nursing human resources.

In recent years, the relationship between case mix index (CMI) and nursing workload in diagnosis-related groups (DRGs) has been explored by scholars. DRGs is a clinical disease diagnosis and treatment classification scheme that was first created and implemented in the United States [8]. The scheme is based on patient diagnosis while taking into account treatment options, complications, severity of comorbidities, age, and other factors. DRG classifies patients with essentially similar diseases and treatment methods, as well as comparable medical resource consumption, into the same group to establish a system of multiple diagnostic groups for management [9–11]. CMI is based on DRGs, and is calculated based on the weighted mean of each group. It is a quantification of the difficulty of cases admitted to the hospital used to reflect the skills of the hospital and clinical complexity of the inpatients [12–15]. CMI may serve as a sensitive indicator of the severity of patient’s conditions and required nursing resources [16]. Studies have shown that the CMI of different departments reflected their different nursing workloads, and that CMI was positively correlated with nursing workload [16]. Therefore, we propose that a higher CMI corresponds to a higher nursing workload and thus a higher number of required nurses.

However, the nursing worktime is the most basic method of determining nursing workload for a long time [1]. Its accurate measurement requires standardized operation support, a large number of manpower, and a long period of measurement. Many previous studies have used self-timing to measure the nursing worktime [17, 18], which may yield different results for the same nursing operation due to the nurses’ differing abilities and non-standardized operations, resulting in unreliable measurements. We are concerned that CMI can be easily and accurately extracted through the hospital information system (HIS), and is closely related to nursing workload, so we are trying to use it for allocating nursing human resources. But the quantitative relationship between CMI and nursing worktime has not been revealed. Therefore, in this study, nursing operation training was conducted in strict accordance with standardized procedures, and the mean time spent by nursing staff of different levels for each nursing operation item was used to derive relatively accurate and reliable nursing hour data. This allows for the establishment of a more credible quantitative relationship between CMI and nursing worktime so as to provide a basis for scientifically sound and convenient allocation of nursing human resources.

## Materials and methods

### Subjects

According to the principle of convenient sampling, we took the patients of hepatobiliary surgery as an example to study. The diseases treated in the Department of hepatobiliary surgery cover 89 DRGs, with many kinds of related nursing operation items (involving a total of 79 items), and covering representative operations of both medical and surgical nursing.

To fully reflect the nursing workload of the department of hepatobiliary surgery, the inclusion criteria of patients were as follows: All patients admitted to the department of Hepatobiliary Surgery during the study period, and the nursing worktime was calculated according to their actual hospitalization days; with or without surgery; Emergency or elective surgery. No patients were excluded out of the study.

Two hundred and seventy-one inpatients of the department of hepatobiliary surgery were prospectively and consecutively included from August 1 to September 11, 2022, and their CMI values at discharge were extracted from the HIS. Thirty-six nursing staff members from across the department were included during the same period to measure daily nursing worktime (DNW), including the two categories of direct and indirect DNW. To accurately measure direct DNW, four post levels were determined according to nurses’ education levels, job titles, and years of work experiences, referring to the recommendations of the Chinese nursing management system [16]; Two nurses from each post level were randomly selected (a total of eight nurses from four post levels) to participate in the measurement of nursing worktime for each direct nursing item.

### Measurement of nursing worktime

#### Establishment of a list of nursing operation items

A literature search was performed by searching the CNKI, Wanfang, and Pubmed databases using keywords including “nursing worktime”, “nursing hours”, “nursing human resource allocation”, and “nurse staffing.” The nursing items obtained were combined with clinical experience with reference to the HIS to initially formulate the framework of nursing items. A *Nursing hour measurement scale of the department of hepatobiliary surgery* was then established using the Delphi method. After two rounds of consultation with 12 experts, the level of authority (consistency ratio, Cr) of the experts reached 0.90. Based on the acceptable reliability criteria (Cr ≥ 0.70 [19]), 79 nursing items were ultimately determined, divided into two categories: direct and indirect nursing items. Direct nursing items referred to actions in which nurses provided direct patient care at the patient’s side [20] and included a total of 64 items in 4 dimensions (as shown in Appendix Table 1-A). These included 41 items related to basic care (e.g., morning and evening care, various patient assessments, oral care, pressure ulcer care, nebulized inhalation, intravenous infusion, etc.), nine items related to specialty care (e.g., maintenance of deep venous catheter, measurement of abdominal circumference, stoma care, etc.), seven items related to perioperative care (e.g. skin preparation, surgical drain care, recording of access volume, etc.), and seven items related to safety education (e.g., admission and discharge education, perioperative education, informed consent for nurse-patient communication, etc.). Indirect nursing items were actions not directly provided for patients but performed in preparation for direct care [20], as well as those required for communication and coordination, and included a total of 15 items (as shown in Appendix Table 1-B). Indirect care items included nursing record writing, execution of computerized medical orders, shift handover, medication preparation, and pre-infusion preparation, among others.

#### Development of the *guidebook for the measurement of nursing hours*

A *Guidebook for the Measurement of Nursing Hours* was formulated based on the basic nursing routine, specialized nursing routine, nursing technical operation specifications, and nursing quality control standards to include the standardized operation procedures of each nursing operation, and the start and end times of each operation care hour measurement, as well as recording methods and precautions, etc.

#### Organization of training and quality control

Training was provided to 36 nursing staff members of the department of hepatobiliary surgery, including a detailed explanation of the purpose of the study, the study methodology, and the main contents of the *Guidebook for the Measurement of Nursing Hours.* A wall clock with a second hand and copies of the *Nursing Hour Measurement Record Form* were distributed. The direct DNW (DDNW) measurement was performed based on the standardized operation of eight nurses and data extraction from the HIS, which were less likely to be influenced by individual factors. The measurement of indirect DNW (IDNW) was done using self-timing, which may be subject to inaccuracy due to individual operation and timing methods and lead to discrepancies in the results. Therefore, before the formal measurement, a one-week premeasurement of indirect nursing worktime was performed to predict the volume in order to equip observers and surveyed nursing staff with specific measurement methods and answer questions about the use of the *Guidebook for the Measurement of Nursing Hours*.

During the premeasurement period, all questionnaires from the previous day were collected at around 8:30 a.m. each day. Any doubts about the data were immediately discussed and verified with the specific measurement personnel, and improvements were made in a timely manner to ensure data accuracy.

#### Measurement of DDNW for patients

Standardized operations for 64 direct nursing items were performed by eight selected nursing staff to accurately record the operation time. For direct nursing operations that required multiple nursing staff to work together, the measured time was multiplied by the number of nurses to obtain the total nursing worktime. The mean operating time of eight nursing staff for each direct nursing item was taken as mean nursing worktime per direct nursing item. The frequencies of the direct nursing items were extracted from the medical order system, billing system, and nursing records of the HIS database (DDNW = ∑ (mean nursing worktime per direct nursing item × frequency of this direct nursing item during hospitalization) / total number of days the patient was hospitalized).

#### Measurement of IDNW for patients

The Total indirect nursing worktime were measured using self-timing, with 36 nurses performing the measurement for one consecutive week between August 15 and August 21, 2022. Measurement was performed on day shifts, mid-shifts, and night shifts (IDNW = sum of all indirect nursing worktime during the measurement period / total hospital stay of all patients during the measurement period).

### Establishment of the quantitative relationship between DNW and CMI

#### Calculation of DNW

We defined DNW was the sum of the DDNW and IDNW.

#### Analysis of the factors affecting the relationship between DNW and CMI

In China, graded nursing was implemented in 1956 by Kaixiu Zhang and Xiufang Li [17], and remains in place to this day. In this study, primary, secondary, and tertiary care were assigned scores of 4, 2, and 1, respectively. The daily care scores were summed and divided by the hospital days as the average level of care for that patient. An analysis of covariance was performed to observe the relationship between CMI and DRGs while adjusting for three confounders, namely, patient gender, age, and average level of care.

#### Establishment of the quantified relationship between DNW and CMI

The 10 bivariate relationship models of curve estimation in the SPSS software were used to analyze the quantitative relationship between DNW and CMI. The equation with the highest coefficient of determination and with all regression coefficients being significant was chosen as the optimal equation for the quantified relationship.

### Assessment of applicability of the quantitative relationship model between DNW and CMI

Seventeen clinical departments, including the department of hepatobiliary surgery, were retrospectively included. The CMI of these departments were extracted from the HIS to calculate DNW based on the model established above. Then the total number of beds in each department and the bed utilization rates were extracted to derive theoretical nursing human resource allocation according to the following formula, so as to calculate the relative gap between theoretical and actual nursing human resources. Finally, the rationality of the nursing human resource allocation in each department was assessed based on the relationship between the relative gap and patient satisfaction, as well as the incidence of adverse events.

Based on DNW calculation method recommended by literature [21], we calculated nursing human resource allocation in clinical departments according to the following modified formula: (CMI corresponding to daily nursing hours per patient × total number of beds in the department × bed utilization rate in the department × mobility factor × rest factor) / daily nursing hours per nurse).

Here, the CMI uses average monthly or yearly CMI values for clinical departments, instead of the patient CMI values.

Mobility factor refers to the increase in the number of staff on top of the general official staffing due to normal absences (including leave, maternity leave, study trips, sick leave, etc.) [17]. In this study, the mobility factor was set at 1.2 based on the *Trial Draft of the Organizational Principles for General Hospitals* formulated by the former Ministry of Health in 1978, which used a mobility factor of 20–25% [17].

Rest factor is the ratio of calendar days to legal working days. The number of normal vacation days per year for nurses includes 2 days of sabbatical leave per week (52 weeks per year) and 11 days of legal holidays, which adds up to a total of 115 days. The rest factor was calculated as 365/ (365–115) = 1.46.

The desirable unit value of nursing hours was reported to be an average of 45 min effective work time per hour [17]. Therefore, the daily nursing hours per nurse was projected to be 6 h for an 8 h shift.

### Statistical analysis

SPSS 26.0 was used for statistical analysis. By the Shapiro-Wilk test, measures that met the normal distribution were expressed as mean ± standard deviation ($\overline{x}$ ± s), and those that met the non-normal distribution were described using M (P25, P75). The effects of gender, age, DRGs, and level of care on the relationship between DNW and CMI were analyzed using covariance. Curve estimation was selected to establish the optimal quantitative relationship model between DNW and CMI. Pearson correlation and partial correlation were also used to analyze the association between nursing human resource allocation gaps and patient satisfaction as well as with adverse event rates to assess the rationality of nursing human resource allocation. A significance level of 0.05 was adopted.

## Results

### Basic subject information

Thirty-six nurses aged 20 to 51 years old, with a mean age of 30.2 ± 7.9 years. They had 1 to 31 years of experience, with a median (P25, P75) of 8.5 (2.3, 12.0) years. Their educational background composition was 91.7% (33/36) for bachelor’s and 8.3% (3/36) for college degrees. Their nursing job levels were 16.7% (6/36), 25% (9/36), 27.8% (10/36), and 30.6% (11/36) for post level of A, B, C, and D, respectively.

Two hundred and seventy-one patients were included in this survey. Among them were 167 males and 104 females, aged 22 to 91 years with a median (P25, P75) of 53(47, 62.3) years. According to ICD-10, the patients were in 89 DRGs, with the top 5 most common conditions in descending order being laparoscopic cholecystectomy without complications (ICD-10419), pancreatic and hepatic bypass with severe MCC (multiple chronic conditions) (ICD 10405), disorders of the biliary tract without CC/MCC (ICD-10446), laparoscopic cholecystectomy with complications (ICD-10418), and pancreatic and liver bypass without complications (ICD-10407). Their CMI values ranged from 0.49 to 4.17, with a median (P25, P75) of 2.62 (0.92, 4.07).

### Indirect and direct DNW and percentages

**Fig. 1:**
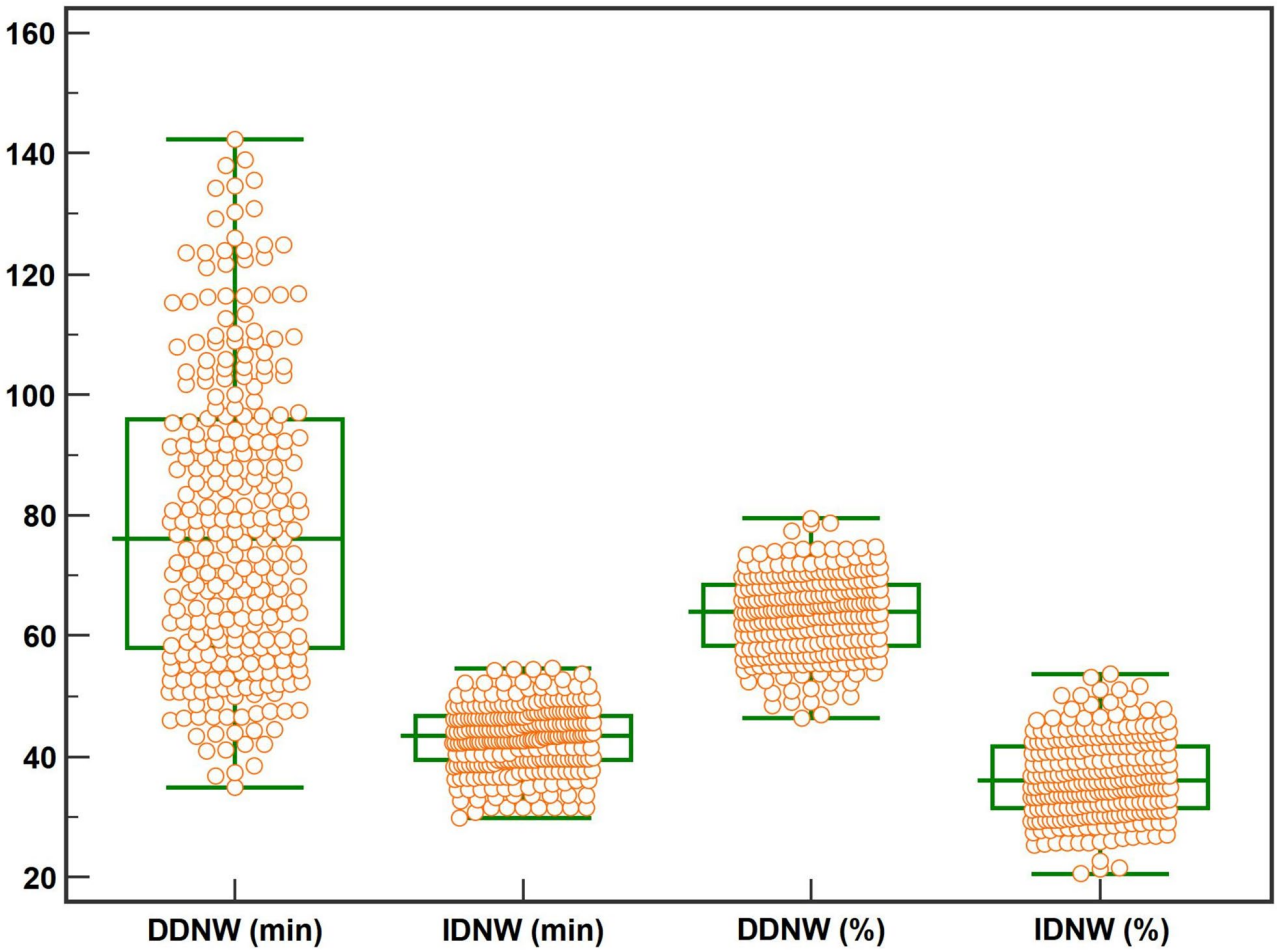
The Box-Whisker plot of the direct and indirect DNW percentage in 271 patients. Note: DNW, daily nursing hours

A Box-Whisker plot of the average DDNW and IDNW and their percentages was made (**see** Fig. 1). The results showed that the average DDNW and its percentage were 76.1 (58.0, 98.9) min and 64.0% (58.3, 68.5%), respectively. The average IDNW and its percentage were 43.42 (39.42, 46.72) min and 36.0% (31.5, 41.7%), respectively.

### Effect of gender, age, DRGs, and level of care on the relationship between DNW and CMI

The results of the analysis of covariance showed that the relationship between DNW and CMI was significantly different between DRGs (*F* = 3028.456, *P* < 0.001) but was not affected by the main individual factors, namely gender (*F* = 0.481, = 0.488), age (*F* = 2.922, *P* = 0.089), and level of care (*F* = 0.096, *P* = 0.757). These findings suggest that different CMIs reflected the nursing workloads of different DRGs and took into account multiple individualized patient factors. Therefore, CMI can be used as the main indicator for nursing workload.

### Construction of the quantitative equations between DNW and CMI

**Fig. 2:**
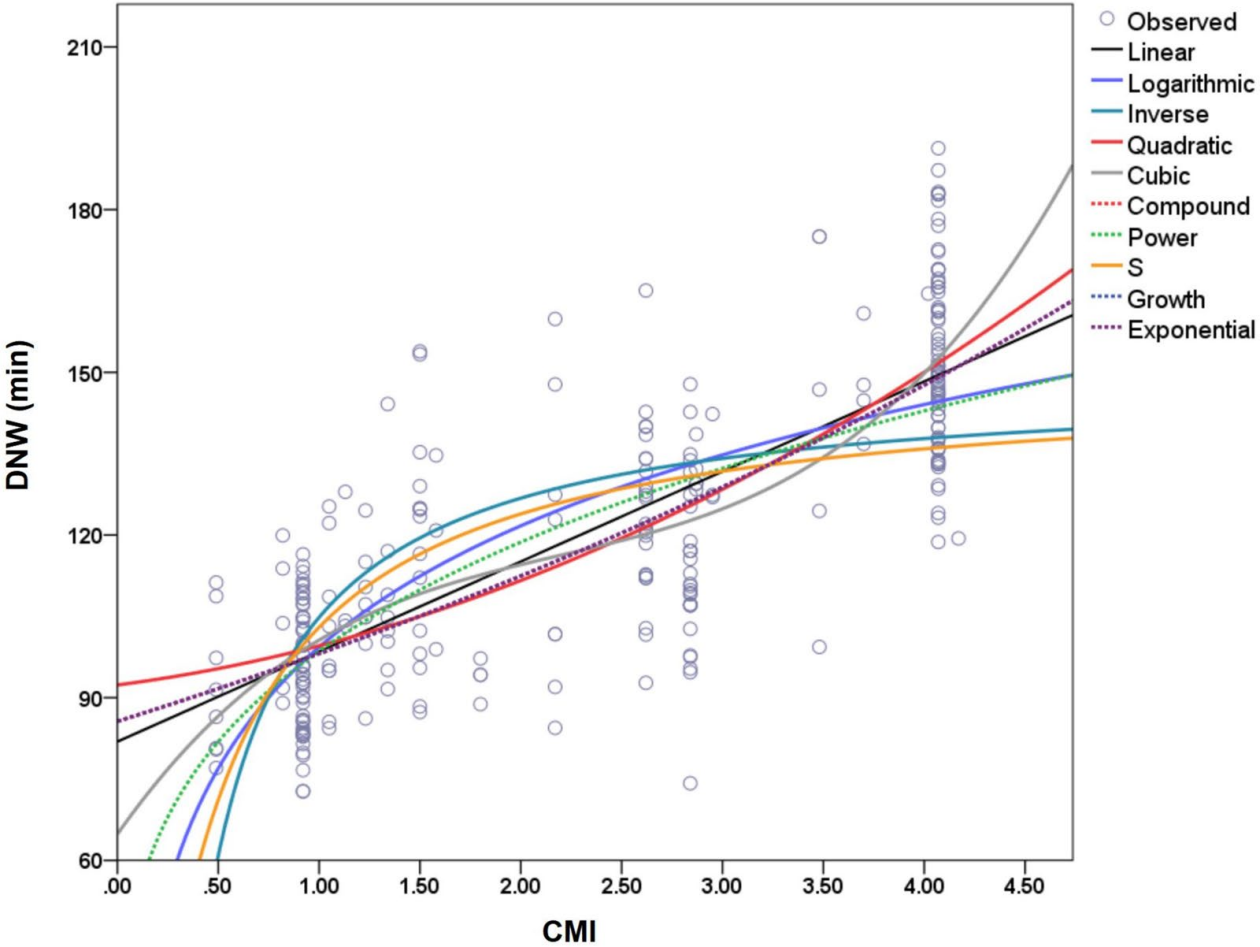
The curvefit between DNW and CMI. Note: DNW, daily nursing hours. CMI, Case Mix Index. A quadratic equation can establish the best quantitative relationship between DNW and CMI, with optimal goodness of fit *R*^2^ = 0.627, and credible regression coefficients (quadratic coefficient: *t* = 2.552, *P* = 0.011; monomial coefficient: *t* = 2.141, *P* = 0.033)

**Fig. 3:**
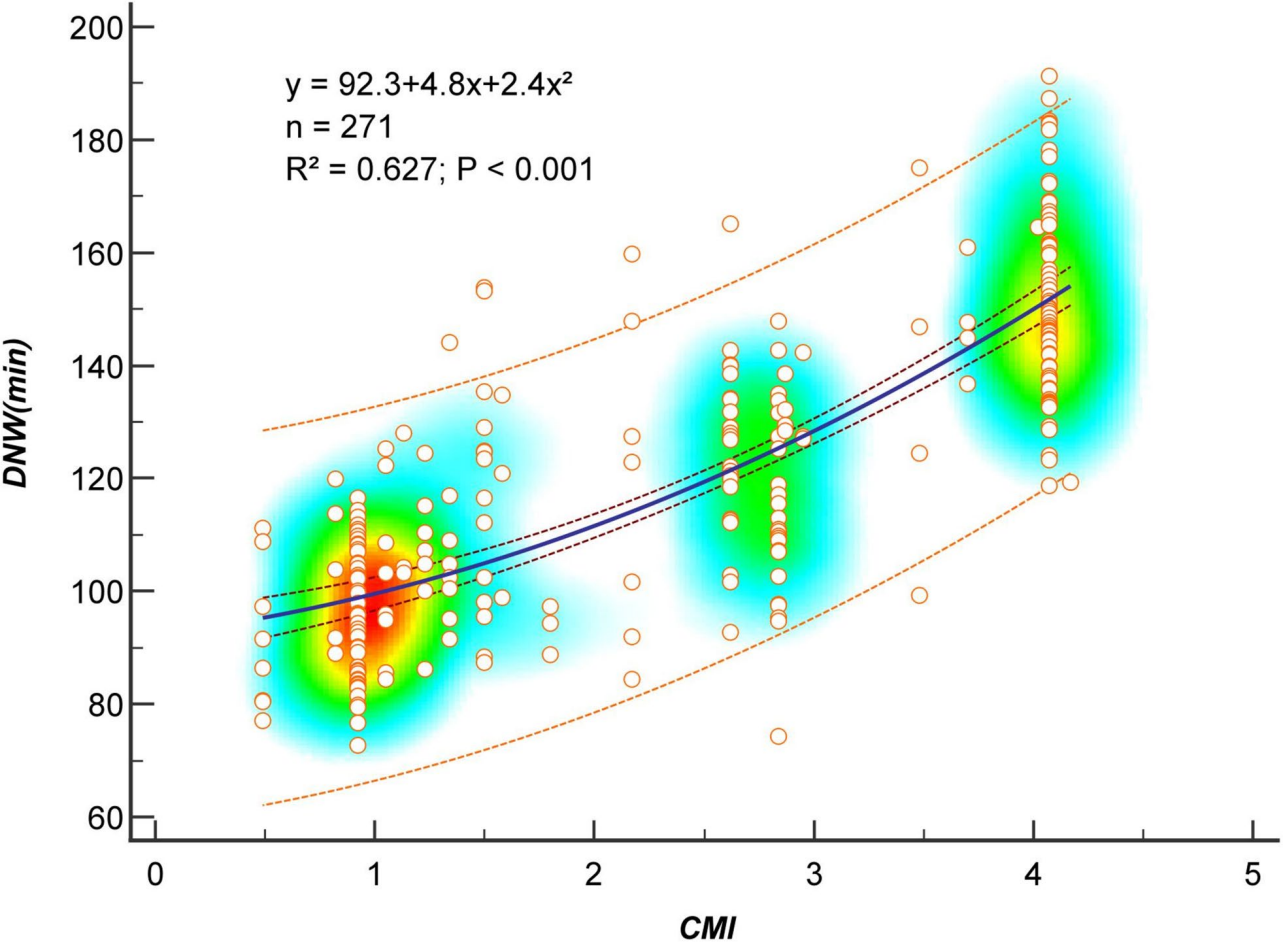
The optimal curvefit between DNW and CMI. Note: DNW, daily nursing hours. CMI, Case Mix Index. The two dashed lines adjacent to the regression curve represent the 95% confidence interval for the regression line; the two dashed lines away from the regression curve represent the 95% prediction interval for the regression curve. Heat map, its background color coding indicates density of data points, suggesting clusters of data observations

The curve estimation results (see Fig. 2 and Table 1) showed that the top three coefficients of determination (R^2^) were cubic (0.636), quadratic (0.627), and linear (0.618) equations, in descending order. The test of regression coefficients showed that, except for the quadratic term of the cubic equation that was not significant (*t* = − 1.108, *P* = 0.269), the regression coefficients of the other terms in the cubic equation (cubic term: *t* = 2.436, *P* = 0.015; first power term: *t* = 2.637, *P* = 0.009), the quadratic equation (quadratic term: *t* = 2.552, *P* = 0.011; first power term: *t* = 2.141, *P* = 0.033), and the linear equation (*t* = 20.904, *P* < 0.001) all met the requirements for establishing the equation. Based on the results, the quadratic model established the optimal quantitative relationship between DNW and CMI (*R*^2^ = 0.627, *F* = 225.1, *P* < 0.001, **see** Fig. 3).

**Table 1:** Equations of 10 curve estimation models between DNW and CMI

Equation model	Formula	*R*^2^	*F*	*P*
Linear equation	*y* = 81.9 + 16.6*x*	0.618	434.6	< 0.001
Quadratic equation	*y* = 92.3 + 4.8*x* + 2.4*x*^2^	0.627	225.1	< 0.001
Cubic equation	*y* = 64.8 + 52.8*x* − 20.1*x*^2^ + 3.0*x*^3^	0.636	155.5	< 0.001
Logarithmic equation	*y* = 99.3 + 32.3 log(*x*)	0.568	354.1	< 0.001
Reciprocal equation	*y* = 148.9 − 44.0/*x*	0.455	224.9	< 0.001
Exponential equation	*y* = 85.6 exp(0.14*x*)	0.617	433.5	< 0.001
Growth curve	*y* = exp(4.45 + 0.14*x*)	0.617	433.5	< 0.001
Composite curve	*y* = 85.6 + 1.14^*x*^	0.617	433.5	< 0.001
Power function	*y* = 98.6*x*^0.27^	0.581	373.7	< 0.001
S-curve	*y* = exp(5.00 − 0.37/*x*)	0.479	246.8	< 0.001

The y stands for DNW (daily nursing worktime), and the x stands for CMI (case mix index)

### Assessment of the nursing human resource allocation model

Information regarding CMI, total number of beds, bed occupancy rate, theoretical and actual number of nursing staff, relative shortage of nursing staff, patient satisfaction, and incidence of adverse events in 17 clinical departments are shown in Table 2**.** Pearson correlation analysis results showed that the relative gap in nursing staff was positively correlated with patient satisfaction over the same period (*r* = 0.653. *P* = 0.006) and negatively correlated with the incidence of adverse events (*r* = − 0.567. *P* = 0.021). In a multivariate partial correlation analysis adjusting for CMI, number of beds, and bed occupancy, the relative shortage of nursing staff was still positively associated with patient satisfaction (*r*_*partial*_ = 0.636. *P* = 0.026) but not correlated with the incidence of adverse events (*r*_*partial*_ = 0.106, *P* = 0.744).

**Table 2:** Related data of nursing human resource allocation and model evaluation in 17 departments

Departments	CMI	Number of bed	Bed rotation rate (%)	Theory staffing (persons)	Actual staffing (persons)	Relative gap (%)	Patient satisfaction (%)	Adverse event (%)
Cardiothoracic Surgery	2.92	56	84	29	24	−17	91.05	0.173
Urinary Surgery	1.26	60	96	29	23	−20	92.1	0.371
Endocrinology	0.78	28	104	14	19	38	95.41	0.047
Neurosurgery	2.97	66	85	35	31	−11	91.33	0.471
Neurology	1.04	110	90	48	47	−2	90.8	0.028
Orthopedics	1.2	30	96	14	14	−2	92.5	0.137
Gastrointestinal Surgery	1.45	108	90	49	42	−15	91.7	0.165
Traditional Chinese	1.12	42	86	18	12	−32	91.2	0.339
Cardiovascular	1.89	115	93	57	49	−14	90.22	0.683
Orthopedics	1.97	85	95	44	46	5	92.47	0.137
Hepatological Surgery	1.43	78	97	38	30	−22	90.33	0.333
Vascular Surgery	1.54	33	84	14	16	13	96.1	0.066
Ophthalmology	0.62	51	88	21	20	−5	97.49	0.122
Otolaryngology	0.83	57	109	30	23	−22	90.75	0.230
Nephrology	1.05	70	93	32	28	−12	89.99	0.217
Gastroenterology	1.03	88	110	47	38	−19	90.23	0.430
Hematopathology	1.52	55	115	32	35	9	92.3	0.133

Theoretical staffing is calculated according to the CMI-based nursing human resource allocation estimation formula. CMI corresponding to DNW per patient × total number of beds in the department × bed utilization rate in the department × mobility factor × rest factor) / daily nursing hours per nurse. Relative gap = (Actual configuration - Theoretical configuration)/Theoretical configuration

## Discussion

At present, studies have demonstrated the positive correlation between CMI and DNW, but they have not explored the quantitative relationship between the two [16].Due to the limitation of administrative responsibilities, our study established the second-order linear quantitative relationship between patients’ CMI and nurses’ DNW for the first time based on the principle of convenient sampling, which provided a feasible means to conveniently convert nurses’ DNW from patients’ CMI, and was conducive to the effective evaluation of nurses’ human resources allocation in the department of hepatobiliary surgery. Fortunately, our research results have been widely applied to the evaluation of human resource allocation of most medical and surgical nurses, and have obtained satisfactory results, the research results may have some popularization value, and open a convenient door for the nursing staff allocation of most departments in hospitals. Convenient staffing assessment can promote administrative functional departments to rationally arrange the effective flow of nursing staff, reduce the excessive waste of human resources for some departments, and reduce the risk of medical safety accidents caused by insufficient human resources for other departments, which has an important positive role in the reform of nursing staff management. In the future, the hospital can directly extract the CMI value of the patient through HIS system, and automatically calculate the number of nursing staff to be allocated through the formula, so that the number of nursing staff can be more convenient and scientific.

### A credible DNW, the key indicator of nursing human resource allocation, may be easily available from CMI

Currently, many studies are using DNW to directly allocate the numbers of nursing staff. However, the measurement of DNW is complicated, time-consuming, and labor-intensive. Moreover, DNW may change with the different types of diseases of that admitted patients might have, making it difficult to reflect the actual number of nursing human resources required. CMI dynamically changes with the types of diseases of the admitted patients and the varying amount of health care resources therefore consumed. A higher CMI is associated with an increase in DNW due to the fact that DRGs are classified according to the patients’ consumption of health care resources. Patients with high CMIs have an increased consumption of health care resources and tend to have a higher demand for nursing human resources [16]. This study explored the quantitative relationship between CMI and DNW, and found that CMI may be used to easily access a credible DNW, so as to allocate human resources of nursing staff in the department of hepatobiliary surgery. Moreover, the data needed to calculate CMI can be extracted directly from the HIS, which is simple and convenient to operate.

### Nursing human resources may be revitalized through the dynamic adjustment of the number of nursing staff based on CMI

On the first day of each month, the hospital can announce the CMI of each department from the previous month. The nursing department can then dynamically adjust the number of nursing staff that should be allocated on top of the fixed number of nursing staff in the following month based on the department’s CMI in the previous month. For example, when the CMI decreases, the number of nursing staff needed would decrease, and the excess nursing staff in the department of hepatobiliary surgery can be temporarily deployed to other departments. When the CMI increases, and in the event of a shortage of nursing staff, the nursing department can transfer staff from other departments in the hospital to the department of hepatobiliary surgery. This dynamic adjustment of nursing human resources may avoid relative deficiencies and waste as well as effectively meet patient care requirements to further ensure nursing safety.

### Further improvement of the hospital support system to improve nursing efficiency

Our data showed that the total DNW was 121.6 minutes, of which 78.7 minutes or 64.7% was direct DNW and 42.9 minutes or 35.3% was indirect DNW. In this study, nursing document writing and drug preparation accounted for a relatively large proportion of indirect DNW. With the popularization and improvement of hospital information technology, the time spent on nursing documentation has been reduced to a certain extent. However, due to the specialization of nursing, specialized assessment forms such as the venous thromboembolism (VTE) risk assessment, stress injury risk assessment, self-care assessment, catheter dislodgement risk assessment, and other nursing forms required nurses to make daily assessments corresponding to the criteria. The hospital information system could not automatically generate assessment recommendations based on connections between the patient’s relevant test indicators and the assessment forms, which extended nurses’ time spent on documentation to a certain extent. It is recommended that the hospital strengthen the information technology construction to be able to link the patient’s indicators with the nursing assessment forms so as to facilitate nurses’ ability to make accurate and fast condition assessments and shorten the time needed for writing nursing documentation. Secondly, the infusion rate of the department of hepatobiliary surgery was as high as 86.89%, with an average number of infusion groups per patient of 4–6 bottles. Furthermore, the hospital did not implement centralized medication distribution, and the clinical nurses prepared the drugs themselves. It is suggested that the infusion rate of patients be further reduced, and that the hospital set up a centralized medication distribution center to allow nurses to devote more time to direct nursing care, such as observation of patients’ special conditions and health education.

### The CMI-based nursing human resource allocation model had certain generalizability

This study showed that the relative gap in nursing staff had a strong positive correlation with patient satisfaction, which was consistent with the study by Twigg et al [2] This was mainly because when there was a shortage of nurses, the time taken for communication, education, and answering questions would be significantly reduced, which was highly likely to lead to patient dissatisfaction. This relative gap was also correlated with the incidence of adverse events of nursing. However, the correlation between nursing adverse events and CMI was found to be stronger when controlling for some of the influencing factors, suggesting that the onset of adverse events was not related only to insufficient nursing human resources, but was more likely to be related to the severity of patients’ conditions and difficulty of care. Therefore, the model truly reflected the number of nursing staff required by different departments and was scientifically sound and generalizable to a certain extent.

### Limitations

This study was not specific with the medication distribution time in terms of the number of drugs dispensed per patient in indirect DNW. However, this data had little impact on the indirect DNW measurement throughout their hospitalization period. Additionally, this study was limited to the department of hepatobiliary surgery, and there were limitations in the included CMI and measured DNW. Multi-specialty and multi-center studies should be subsequently conducted to make the nursing human resource allocation model more generalizable.

## Conclusions

This study established the quantitative relationship between CMI for hepatobiliary surgery and nurses DNW for the first time. Its applicability to the allocation of nursing human resources was verified in most clinical departments. CMI may serve as a sensitive indicator of the severity of patient’s conditions and required nursing resources. Moreover, the CMI of each patient undergoes dynamic changes in response to disease severity and resource utilization. Therefore, allocating nursing human resources based on CMI fully embodies the patient-centered concept and facilitates the dynamic adjustment of nursing staff. It may have a positive effect on the reform of nursing manpower management, and may promote the transformation of human resource allocation from “fixed allocation of beds or departments” to “patient-centered dynamic allocation”.

### Supplementary Information

**Additional file 1.**

**Additional file 2.**

## Data Availability

The datasets used and/or analysed during the current study are available from the corresponding author on reasonable request.

## Abbreviations

CMI: Case mix index
DNW: Daily nursing worktime
DDNW: Direct DNW
IDNW: Indirect DNW
DRGs: Diagnosis-related groups
HIS: Hospital information system

## References

[1] Chen H, Zhao S, Feng L (2016). Decision support system of nursing human resources allocation in general wards based on hospital information system. Stud Health Technol Inform.

[2] Gunawan J, Aungsuroch Y, Fisher ML (2019). Competence-based human resource management in nursing: a literature review. Nurs Forum.

[3] Twigg D, Duffield C, Bremner A, Rapley P, Finn J (2011). The impact of the nursing hours per patient day (NHPPD) staffing method on patient outcomes: a retrospective analysis of patient and staffing data. Int J Nurs Stud.

[4] Griffiths P, Ball J, Drennan J, Dall'Ora C, Jones J, Maruotti A, Pope C, Recio Saucedo A, Simon M (2016). Nurse staffing and patient outcomes: strengths and limitations of the evidence to inform policy and practice. A review and discussion paper based on evidence reviewed for the National Institute for health and care excellence safe staffing guideline development. Int J Nurs Stud.

[5] Driscoll A, Grant MJ, Carroll D, Dalton S, Deaton C, Jones I, Lehwaldt D, McKee G, Munyombwe T, Astin F (2018). The effect of nurse-to-patient ratios on nurse-sensitive patient outcomes in acute specialist units: a systematic review and meta-analysis. Eur J Cardiovasc Nurs.

[6] Adomat R, Hewison A (2004). Assessing patient category/dependence systems for determining the nurse/patient ratio in ICU and HDU: a review of approaches. J Nurs Manag.

[7] Wang YL, Zhou SX, Wu WH (1996). Feasibility study of using the improved PRN in clinical nursing care (project of research in nursing). Zhonghua Hu Li Za Zhi.

[8] Chen YJ, Zhang XY, Yan JQ, Xue-Tang QMC, Ying XH (2023). Impact of diagnosis-related groups on inpatient quality of health care: a systematic review and Meta-analysis. Inquiry.

[9] Zou K, Li HY, Zhou D, Liao ZJ (2020). The effects of diagnosis-related groups payment on hospital healthcare in China: a systematic review. BMC Health Serv Res.

[10] Palmer G, Reid B (2001). Evaluation of the performance of diagnosis-related groups and similar casemix systems: methodological issues. Health Serv Manag Res.

[11] Woo JL, Anderson BR (2020). Diagnosis-related groups, reimbursement, and the quality disconnect. World J Pediatr Congenit Heart Surg.

[12] Joya RM, Cottrell L, Kiefer A, Polak MJ (2022). Diagnosis-related group weight and derived case mix index to assess the complexity among twins. Am J Perinatol.

[13] Behling KC, Bierl C (2019). Cost per case mix index-adjusted hospital day as a measure of effective laboratory utilization efforts in a growing Academic Medical Center. Am J Clin Pathol.

[14] Aiello FA, Judelson DR, Durgin JM, Doucet DR, Simons JP, Durocher DM, Flahive JM, Schanzer A (2018). A physician-led initiative to improve clinical documentation results in improved health care documentation, case mix index, and increased contribution margin. J Vasc Surg.

[15] Redmann AJ, Yuen SN, VonAllmen D, Rothstein A, Tang A, Breen J, Collar R (2019). Does surgical volume and complexity affect cost and mortality in otolaryngology-head and neck surgery?. Otolaryngol Head Neck Surg.

[16] Han B, Chen X, Li Q (2018). Application of case mix index in the allocation of nursing human resources. J Nurs Manag.

[17] Ji YY, Wang J, Zheng DG, Teng D, Wang N, Liu YY (2020). Research on allocation of nursing human resource in neurosurgery department based on working time measurement and nursing grading. Chin Nurs Res.

[18] Min A, Scott LD (2016). Evaluating nursing hours per patient day as a nurse staffing measure. J Nurs Manag.

[19] Rodríguez-Suárez CA, Rodríguez-Álvaro M, García-Hernández AM, Fernández-Gutiérrez DÁ, Martínez-Alberto CE, Brito-Brito PR (2022). Use of the nursing interventions classification and Nurses' workloads: a scoping review. Healthcare.

[20] Kakushi LE, Evora YD (2014). Direct and indirect nursing care time in an intensive care unit. Rev Lat Am Enfermagem.

[21] Twigg D, Duffield C (2009). A review of workload measures: a context for a new staffing methodology in Western Australia. Int J Nurs Stud.

