# Supplementary material for: Construction and application of a nursing human resource allocation model based on the case mix index
Source: BMC Nurs. 2023 Dec 6;22:466. doi: 10.1186/s12912-023-01632-y (PMC10698983; doi:10.1186/s12912-023-01632-y)
Supplement: Supplementary file 1 — Additional file 1. [file 12912_2023_1632_MOESM1_ESM.docx]

**Appendix Table 1. Operation frequency collection table of direct nursing items in Department of hepatobiliary Surgery**

**Name： Bed number： Age： Hospital admission number： Diagnosis：**

| **categories** | **direct nursing items** | **Number** | | |
| --- | --- | --- | --- | --- |
|  |  | **date** | **date** | **date** |
| Basic nursing | Touring the ward |  |  |  |
|  | morning and evening nursing |  |  |  |
|  | Change the sheets |  |  |  |
|  | Patient assessment (falls, pressure sores, self-care ability, VTE, risk of tube loss, psychological, nutritional screening) |  |  |  |
|  | Vital signs measurement (T, P, R, BP, pain) |  |  |  |
|  | Blood glucose monitoring |  |  |  |
|  | physical cooling |  |  |  |
|  | Traditional Chinese Medicine Treatment |  |  |  |
|  | intermediate frequency therapy |  |  |  |
|  | mouth care |  |  |  |
|  | perineal care |  |  |  |
|  | wound care |  |  |  |
|  | Pressure ulcer care |  |  |  |
|  | Nasal feeding therapy |  |  |  |
|  | Urine retention care |  |  |  |
|  | Catheter insertion |  |  |  |
|  | Indwelling catheter extraction |  |  |  |
|  | No retention enema/retention enema/Clean enema/anal tube exhaust |  |  |  |
|  | oral medication |  |  |  |
|  | Subcutaneous/intradermal/intramuscular injection |  |  |  |
|  | Roll over/back pat |  |  |  |
|  | Inhalation therapy |  |  |  |
|  | intravenous injection |  |  |  |
|  | Scalp needle/indwelling needle for intravenous infusion |  |  |  |
|  | The utilization of intravenous infusion pumps |  |  |  |
|  | Peripheral venous catheter nursing |  |  |  |
|  | Intravenous transfusion |  |  |  |
|  | Transporting patients |  |  |  |
|  | adding drugs |  |  |  |
|  | Collecting intravenous blood |  |  |  |
|  | Collecting arterial blood |  |  |  |
|  | Sputum/throat swab specimen collection |  |  |  |
|  | Drainage fluid samples collection |  |  |  |
|  | ECG monitoring |  |  |  |
|  | Bedside handover |  |  |  |
|  | Accompanying the doctor for ward rounds |  |  |  |
|  | Bedside electrocardiogram |  |  |  |
|  | CPR(cardio-pulmonary resuscitation) |  |  |  |
|  | aspiration of sputum |  |  |  |
|  | Tracheotomy nursing |  |  |  |
|  | death nursing |  |  |  |
| specific nursing | PICC/ Midline catheter insertion |  |  |  |
|  | Central venous catheter nursing |  |  |  |
|  | Measure abdominal circumference |  |  |  |
|  | ICG |  |  |  |
|  | Nursing of patients with multi-drug resistant bacteria |  |  |  |
|  | The ostomy bag is placed |  |  |  |
|  | Enterostomy care |  |  |  |
|  | Gastrointestinal tube/triple lumen tube insertion |  |  |  |
|  | Gastrointestinal tube/nasobiliary duct extraction |  |  |  |
| Perioperative nursing | Skin preparation |  |  |  |
|  | Intradermal sensitivity test |  |  |  |
|  | Surgical handover |  |  |  |
|  | Nasal catheter/mask oxygen |  |  |  |
|  | Drainage tube nursing routine |  |  |  |
|  | Drainage replacement |  |  |  |
|  | Recorded output |  |  |  |
| safety education | hospitalization health educatio |  |  |  |
|  | Discharge health education |  |  |  |
|  | Perioperative health education |  |  |  |
|  | Health education (disease related, dietary guidance, medication, examination, activity, psychology) |  |  |  |
|  | Security risk investigation |  |  |  |
|  | Sign informed consent |  |  |  |
|  | admission and discharging |  |  |  |
