# Supplementary material for: Construction and application of a nursing human resource allocation model based on the case mix index
Source: BMC Nurs. 2023 Dec 6;22:466. doi: 10.1186/s12912-023-01632-y (PMC10698983; doi:10.1186/s12912-023-01632-y)
Supplement: Supplementary file 2 — Additional file 2. [file 12912_2023_1632_MOESM2_ESM.docx]

**Appendix Table 2. Operation frequency collection table of** **indirect nursing items in Department of hepatobiliary Surgery**

**Name： Bed number： Age： Hospital admission number： Diagnosis：**

| **indirect nursing items** | **Number** | | |
| --- | --- | --- | --- |
|  | **date** | **date** | **date** |
| Billing/reconciliation of medical orders |  |  |  |
| PDA carried out medical orders |  |  |  |
| Writing nursing documents (nursing records, temperature sheets, shift records) |  |  |  |
| Interpretation charge |  |  |  |
| answer the question |  |  |  |
| Collate medical records |  |  |  |
| nursing consultation |  |  |  |
| carried out Critical value |  |  |  |
| Preparation for infusion/injection |  |  |  |
| Preparation for blood transfusion |  |  |  |
| Preparation before dispensing |  |  |  |
| Nursing handover |  |  |  |
| Patient preparation before examination and surgery |  |  |  |
| Drug Dispensing |  |  |  |
| dispensing medicines |  |  |  |
